# B Cells and Progressive Multifocal Leukoencephalopathy: Search for the Missing Link

**DOI:** 10.3389/fimmu.2015.00241

**Published:** 2015-05-19

**Authors:** Deniz Durali, Marie-Ghislaine de Goër de Herve, Jacques Gasnault, Yassine Taoufik

**Affiliations:** ^1^Immunology Research Laboratory, Department of Medical Microbiology, School of Medicine, Istanbul Medipol University, Istanbul, Turkey; ^2^IMVA-INSERM U1184, Department of Immunology, Bicetre Hospital, University Paris-sud, Le Kremlin-Bicêtre, France

**Keywords:** progressive multifocal leukoencephalopathy, JC virus, B cells, immune regulation, T cells

## Abstract

Progressive multifocal leukoencephalopathy (PML) is a deadly demyelinating disease due to JC virus (JCV) replication in the brain. PML classically occurs in patients with severe immunodepression, and cases have recently been linked to therapeutic monoclonal antibodies such as natalizumab and also rituximab, which depletes B cells. B cells appear to play a complex role in the pathogenesis of PML. They may act as a viral reservoir and as a vector for viral dissemination in the central nervous system. Anti-JCV antibody responses appear to have a limited effect on JCV replication in the brain. However, accumulating evidence suggests that B cells may considerably influence T cell responses through their cytokine secretion. This immunomodulatory function of B cells may play an important role in the control of JCV infection and in the pathogenesis of PML, including rituximab-induced PML.

Progressive multifocal leukoencephalopathy (PML) is a devastating demyelinating disease caused by replication in the brain of the opportunistic polyomavirus JC virus (JCV), which asymptomatically infects a large proportion of the adult population worldwide. PML occurs almost exclusively in patients with severe immunodepression due to disorders such as AIDS, hematological malignancies, and sarcoidosis, but is also a recognized adverse effect of therapeutic monoclonal antibodies such as natalizumab, efalizumab, and rituximab used to treat autoimmune diseases and hematological malignancies (1, 2). Specific CD4 and CD8 T cell responses appear to play a critical role in the control of JCV infection: for instance, the beneficial effect of highly active antiretroviral therapy (HAART) on AIDS-related PML is largely due to restoration of anti-JCV T cell immunity (3–5). The PML-promoting effect of rituximab, an anti-CD20 monoclonal antibody that specifically depletes B cells, suggests that B cells also contribute to the control of JCV infection (1). The incidence of PML in rituximab-treated patients depends on the underlying disease: it is about 2/8,000 in patients with systemic lupus erythematosus (SLE) and 1/25,000 in those with rheumatoid arthritis (RA) (6, 7). B cells have a dual role in PML: first, they can serve as a viral reservoir and may help disseminate the virus in the brain; second, they are an important component of the adaptive immune response and may play a significant role in JCV control.

## B Cells are a Potential JCV Reservoir and a Vector for CNS Dissemination

JC virus infection usually occurs in childhood and persists throughout life. It generally remains clinically silent, despite active virus replication in the kidneys and urinary virus excretion in a significant proportion of the general population. Severe, prolonged immunosuppression may lead to JCV dissemination to the central nervous system (CNS) from sites of persistence (kidney, bone marrow, lymphoid organs), or to reactivation of dormant virus already present in the CNS. In both cases, this may lead to productive infection of oligodendrocytes, followed by demyelination and development of PML (2, 8). Detection of JCV DNA in peripheral B lymphocytes and of JCV-infected B cells in brain tissue of PML patients suggests that B cells are directly involved in JCV dissemination to the CNS (9–12).

JC virus can infect CD34^+^ hematopoietic precursor cells and B cells, but not primary T cells (9, 13). Chapagain et al. showed that JCV can enter B cells and persist as intact virions (12). B cells are probably infected by JCV in lymphoid tissues such as the tonsils, spleen, and bone marrow (14–16). JCV-infected B cells may also derive from latently infected hematopoietic precursors in bone marrow (17–19). Nucleotide sequence analysis of JCV in peripheral blood mononuclear cells (PBMC), urine, and cerebral spinal fluid (CSF) of PML patients has revealed JCV sequence variations and rearrangements that influence viral pathogenicity and tropism (18, 20–25). JCV persists in at least two forms: a non-pathogenic form (archetypal virus) and a neurotropic form that contains a rearranged non-coding control region (NCCR) (20, 24, 26). B cells could serve for the generation, persistence, and dissemination to the CNS of the neurotropic form (27). Glial cells (the main targets of JCV in the brain) and B cells, but not T cells, both express nuclear DNA binding proteins that interact with the regulatory region of the JCV genome and may permit JCV replication (10, 28, 29). The NCCR is involved in transcriptional control of both early and late viral genes (25, 30–33). Two transcription factors (NF-1X and Spi-B) important for JCV genome transcription are upregulated in glial cells, B cells, and hematopoietic progenitor cells (25, 34, 35). Spi-B binding sites are present in the promoter/enhancer of JCV neurotropic variants but not in the archetypal virus. These sites are located in the region adjacent to TATA boxes, which are essential for the transcription of early and late viral genes (35–37). Rituximab modifies B cell homeostasis, and the reconstituted B cell pool after treatment consists mainly of immature (IgD^+^CD10^+^CD24^hi^CD38^hi^) and naive B cells (38–42). Rituximab depletes CD20^+^ mature B cells in the periphery, probably leading to mobilization of pre-B and B cells from bone marrow and lymph nodes, along with an increase in CD34^+^ progenitors in the periphery (17). Infected B cells arising from bone marrow and lymph nodes may transmit the infection to microvascular endothelial cells and, after crossing the blood–brain barrier, to glial cells (11, 12, 14). Also, natalizumab has been reported to inhibit VLA-4-dependent retention of CD34^+^ hematopoietic precursor cells, B cell precursors, and B cells in bone marrow and lymphoid tissues, leading to increased circulation of pre-B and B cells (17, 43). However, it remains unclear how much its effects on B cells may contribute to natalizumab-associated PML.

Thus, the following conditions are required for JCV-induced PML to occur: changes in the NCCR that enhance viral transcription and replication; the presence of transcription factors that bind to the rearranged NCCR; immunodeficiency; and, likely, other factors such as an individual genetic predisposition.

## The Specific Antibody Response Appears Insufficient to Control JCV Infection

Humoral immunity, and particularly the production of neutralizing antibodies, is an important line of defense against viral infections (44). Intrathecal antibody synthesis is observed in infections due to herpes simplex, varicella zoster, Epstein–Barr, cytomegalovirus, mumps, rubella, measles, dengue, and JCVs (45–48). Intrathecal synthesis of oligoclonal antibodies against VP1, the major structural protein of JCV, is found in PML patients, and a positive correlation has been found between the intensity of this response and the plasma cell count in PML brain tissue (45, 49). Between 67 and 78% of PML patients have an anti-VP1 intrathecal antibody response but its protective effect is unclear (45). Intrathecal synthesis of anti-VP1 antibodies with low affinity has occasionally been found in chronic CNS immune disorders such as multiple sclerosis (MS) and neurolupus and infections (mumps meningitis and neuroborreliosis) (50–54). This low-affinity anti-VP1 antibody response may be related to reactivation of memory B cells already present in the CNS (45). T cell and IgG responses to JCV are significantly increased in HIV-infected PML survivors, and the IgG response correlates positively with the CD4 T cell count but negatively with HIV RNA load (55). Neither intrathecal nor serum JCV-specific antibodies prevent the onset or progression of PML in HIV-infected patients (56). A longitudinal study of an HIV-seronegative PML patient showed that the anti-VP1 antibody response increased with time, yet neurological status deteriorated and the patient died (45).

## B Cells Modulate the Differentiation and Functions of CD4 and CD8 T Cells

The use of rituximab to treat autoimmune diseases has provided important clues to the regulatory effects of B cells on cellular immunity. In addition to B cell depletion, rituximab modulates the numbers and functions of peripheral blood lymphocyte subsets such as T, NK, and NKT cells in several autoimmune diseases, including RA, SLE, Evans’ syndrome, and MS (57–61). Rituximab treatment leads to substantial depletion of peripheral T cells, a decrease in the proportion of CD4 cells expressing the early activation marker CD69 and, conversely, an increase of the frequency of CD4^+^CD25^hi^ regulatory T cells (58, 59, 61).

Lykken et al. demonstrated that acute and chronic B cell depletion by an anti-CD20 monoclonal antibody disrupts CD4 and CD8 T cell homeostasis and expansion in mice during acute viral infection (62). B cells appear to be required for optimal CD4 and CD8 T cell responses to acute and chronic viral infections in mice (62, 63). Immunoglobulin mu chain gene knockout (IgM^−/−^ mice) have normal cytotoxic T cell responses to vesicular stomatitis virus (VSV), as well as to vaccinia virus and LCMV (acute Armstrong variant) (63). However, the initially normal CTL response to LCMV infection in IgM^−/−^ mice disappears in the long term, leading to viral persistence (63). Adoptive transfer experiments show that naive and activated antiviral CD8 T cells from transgenic mice expressing an LCMV gp33-specific TCR are rapidly exhausted and disappear after transfusion into mice persistently infected by the LCMV–WE strain (63). Cotransfusion of immune CD4 T cells or primed B cells from infected mice prevents this exhaustion, contrary to transfusion of hyperimmune serum (63). This suggests that the positive effect of B cells on CD8 T cell antiviral functions is independent of antibody secretion. In B-cell-deficient mice, the CD8 T cell response is effective on acute LCMV and influenza virus infection but not on chronic LCMV infection (64–68). The absence of B cells results in increased death of activated CD8 T cells during the contraction phase, leading to poorer antigen-specific CD8 T cell memory (65, 69). CD4 T cells are required for the generation, long-term maintenance, and optimal reactivation of memory CD8 T cells (70–75). B cells are also required for the generation of CD4 T cell memory (68, 76–78). B-cell-deficient IgM^−/−^ mice infected with a persistent LCMV variant have a profound CD4 help defect and secrete less interferon-gamma (IFN-γ) and interleukin 2 (IL-2) than normal mice, a defect mainly affecting CD8 T cells (76). In contrast to B-cell-deficient mice, transgenic mice that have normal proportions of B cells in the periphery but do not secrete LCMV-specific antibodies still have a functional CD4 T cell memory (68). This confirms that the effect of B cells on CD4 T cell memory is independent of antibody secretion. In mice depleted of B cells by anti-CD20 and infected by LCMV (Armstrong strain), primary virus-specific CD4 T cell effectors are generated but the CD4 memory precursor population is reduced and memory T cells show impaired cytokine production (79). These experiments suggest that B cells play a significant role in the generation of CD4 and CD8 T cell memory. As CD4 T cell help is required for CD8 memory T cell generation and maintenance, and as B cells influence CD8 T cell antiviral responses, an indirect effect via CD4 T cells appears likely. The effect of B cells on T cell responses may involve cytokine production (80). Indeed, cytokines secreted by B cells can modulate the differentiation and functions of several immune effectors, including CD4 and CD8 T cells, possibly explaining the antibody-independent immunoregulatory functions of B cells (80–84). The mechanisms that control cytokine production by B cells are therefore drawing increasing attention.

## Effector B Cells as Amplifiers of Th1-Type Responses to Viral Infections

B cells produce cytokines in response to a broad array of stimuli, including microbial products, antigens, and T cell-derived signals (80, 85). Under appropriate conditions *in vitro*, B cells differentiate into effector subgroups 1 and 2 (Be1 and Be2), which produce cytokines associated with Th1 and Th2 responses, respectively (86–89). In mouse experiments, differentiation into Be1 cells is induced by Th1 lymphocytes and mediated by IFN-γ and antigenic activation through B cell receptors (86). Like IFN-γ, IL-12 plays a key role in Be1 polarization, but the initial trigger of Be1 commitment is likely type-I interferons (IFN-α/β) (89, 90). These interferons initiate a cascade of molecular events that induce B cell differentiation into Th1-like cells (89, 90). Similarly, naive B cell differentiation into Be2 cells is dependent on IL-4 (88). Be1 and Be2 cells, by producing polarizing cytokines such as IFN-γ and IL-4, induce the differentiation of naïve CD4 T cells into Th1 and Th2 cells (86). Spatiotemporal interactions between B cells, CD4 T cells, and dendritic cells (DCs) are critical during early viral infection and likely determine the orientation and nature of the immune response. Immediately after VSV infection in mice, antigen-specific B and CD4 T cells interact at the T cell–B cell zone border (91). During initiation of the immune response, intact antigens are presented to B cells by DCs (especially follicular DCs), and then B cells present them in the form of peptides to T cells (92–94). Be1 commitment may be initiated by IFN-α/β and then by IL-12 produced by DCs. After antigen priming, T cells migrate toward the B cell area of lymph nodes where they interact with B cells, which, by secreting Th1-like cytokines, may stabilize Th1 differentiation of CD4 T cells. IFN-γ-secreting Be1 and Th1 cells may positively influence each other, thereby creating a Th1 amplification loop between B and T cells.

As Th1 cells are involved in the control of intracerebral JCV infection (95), the Th1-type amplification loop created by B–T cell interactions might be important for the development of effective anti-JCV immune responses. Withdrawal of natalizumab therapy in multiple sclerosis patients who develop PML leads to an immune reconstitution inflammatory syndrome (IRIS) in the brain, due to massive afflux of autoimmune and JCV-specific T cells (96, 97). In MS patients with PML–IRIS, brain-infiltrating anti-JCV CD4 T cells are largely IFN-γ-secreting cells. Bi-functional Th1-2 cells (secreting both IL-4 and IFN-γ) are also present, while IL-17-producing cells are barely detectable (98). Histopathologic analysis of brain tissue from patients with IRIS has revealed the prominent presence of not only CD4 and CD8 T cells but also B/plasma cells and monocytes (98). The regulation of B cell activation by antigen sequestered within the CNS is unclear. Despite the lack of draining lymphatic vessels in the CNS, antigen-bearing DCs can migrate from the CNS to cervical lymph nodes, preferentially reaching B-cell follicles rather than T cell-rich areas (99). B cells activated by antigen-bearing DCs may interact with T cells and favor Th1 differentiation. Thus, by disrupting Th1 responses (100), rituximab may impair the cellular immune response to JCV.

## B Cells as Regulators of Cellular Immune Responses to Viral Infections

The regulatory effects of B cells on immune responses are complex and not only restricted to Th1- or Th2-like responses: some B cells, described as B regulatory cells (Bregs), also have T regulatory-like activities (80, 101, 102). Besides pro-inflammatory cytokines, many B cell subsets also secrete IL-10, a cytokine that suppresses both the activities of T cells (CD4 and CD8) and innate cell-mediated inflammatory responses, while also being involved in Treg maintenance (81–84, 101–104). Breg function is mainly but not exclusively dependent on IL-10 (80, 101, 102). Mouse and human plasma cells, in addition to their Ig production, could contribute to immune regulation by producing IL-10, like Bregs (105, 106). Interestingly, B cell depletion with rituximab has an inducing effect on Tregs (107–111). In SLE, RA, lupus nephritis, and idiopathic thrombocytopenic purpura patients, and particularly in good responders, the Treg frequency and response are restored or enhanced by rituximab (107–111).

B cell homeostasis is modified after rituximab treatment, and the reconstituted B cell pool consists mainly of immature (IgD^+^CD10^+^CD24^hi^CD38^hi^) and naive B cells with increased CD38 and CD5 expression (112–114). Besides immature and naive B cells, plasma cells are also prominent in the reconstituted B cell population (112, 114). However, CD27^+^ memory B cells recover more slowly than naive B cells and remain below baseline values for about 2 years (112). It has been demonstrated that the cytokine profile (anti- or pro-inflammatory) depends on the B cell differentiation stage (naive, memory, etc.) (113). Indeed, IL-10 is produced almost exclusively by naive B cells, while the pro-inflammatory cytokines lymphotoxin (LT) and tumor necrosis factor (TNF-α) are mainly produced by memory B cells (113). Therefore, rituximab-induced changes in the reconstituted B cell population may also affect the overall B cell cytokine profile (113). Naive B cells predominate in the post-rituximab B cell population; in addition, IL-10 production is enhanced and LT and TNF-α production is downregulated as compared to the pretreatment situation (113). The impact of cytokine changes induced by B cell depletion is evident in myasthenia gravis patients who respond well to rituximab: indeed, these patients exhibit rapid repopulation by IL-10-producing B cells and a sustained increase in the circulating Treg frequency, contrary to non-responders (115, 116). The immunosuppressive effect of rituximab could result from the disappearance of Be1 cells leading to failure of effector T cell activation, and also from the selective survival and repopulation of Breg-like subsets. It has been shown that human CD19^+^CD24^hi^CD38^hi^ B cells have regulatory effects that include inhibition of the differentiation of naive T cells into Th1/Th17 cells and the conversion of CD4^+^ CD25^−^ T cells into Tregs by IL-10 (102, 117). In addition to CD19^+^ B cells, it has recently been found that plasmablasts and plasma cells are important IL-10 producers and that they can inhibit the effects of DCs on the generation of effector T cells (118, 119). In addition to IL-10, plasma cells also produce IL-35 (119). IL-35, which induces Tregs, also regulates the expansion and activity of IL-10-producing Bregs (119–122). Wang et al. have shown that IL-35 induces B cell differentiation into a Breg subset that produces IL-35 as well as IL-10 (122). Mice that lack IL-35 or are defective in IL-35 signaling produce fewer Bregs and develop severe experimental autoimmune uveitis (122). Together, these results suggest that naive B cells, memory B cells, and plasma cells have distinct roles in regulating immune responses by secreting cytokines with pro- or anti-inflammatory effects, and that rituximab treatment can induce a shift toward a regulatory-like cytokine profile. Early during B cell reconstitution after rituximab treatment, the predominant response seems to be Breg-like, while Be1- and Be2-like responses only appear once memory B cells emerge.

**Figure 1:**
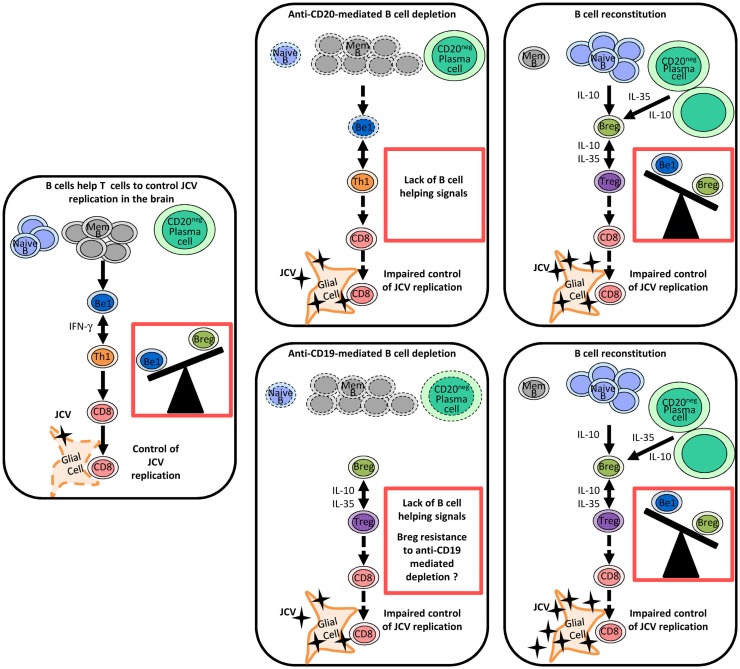
**Regulation of anti-JCV T cell responses by different B cell subsets and the impact of therapeutic B cell depletion on this regulation**. In this model, naive and memory B cells and plasma cells play distinct roles in the regulation of antiviral immune responses through the release of different cytokines. Following therapeutic B cell depletion, there is a shift towards regulatory-like cytokine secretion by the B cell pool. Before therapeutic B cell depletion, IFN-γ-secreting Be1 and Th1 cells mutually enhance each other’s functions and favor a CD8 T cell response, which effectively controls JCV infection. B cell depletion disrupts the Th1 amplification loop and thereby impairs T cell responses to JCV. In contrast to anti-CD20, anti-CD19 depletes also plasma cells. After therapeutic B cell depletion, the B cell pool is mainly reconstituted by naive B cells and plasma cells (IL-10- and IL-35-producing cells), which may promote Treg-like responses. CD1d^hi^ CD5^+^ regulatory B cells may exhibit some resistance to anti-CD19-mediated depletion. Enhanced Breg and Treg responses disrupt T cell-mediated control of JCV infection and may favor the emergence of PML. Abbreviations: Mem B, memory B cell; Be1, effector B cell subgroup 1 (Th1-like B cells); Breg, B regulatory cells (Treg-like B cells); Th1, T helper 1 cells, Treg, regulatory T cells.

The effect of rituximab on B and T cell responses in the CNS is well documented because of the beneficial effects of this drug in MS (123, 124). In particular, rituximab has been shown to deplete B cells in CSF (123, 125–127). In addition, necropsy studies of patients who died of rituximab-induced PML have shown that rituximab also depletes B cells in cerebral perivascular spaces (127). Rituximab could promote the onset of PML by successive effects on B cell homeostasis. First, it eliminates Be1 cells, thereby inhibiting the activation of effector T cells (Figure 1). Then, as shown in Figure 1, repopulation by Breg-like cells such as IL-10-producing B cells and plasma cells, initially in the periphery and then in the CNS, promotes a Treg-like response and inhibits inflammatory responses (81, 128, 129). *In vitro* experiments suggest that Bregs could influence T cell responses in brain via IL-10, by inhibiting microglia activation following viral antigen stimulation and promoting Treg proliferation (128). It remains to be determined whether B cell-depleting antibodies other than anti-CD20 have the same potential to induce PML. In the EAE model, a single injection of monoclonal anti-CD19 inhibited leukocyte infiltration into the spinal cord and disrupted disease development (130). In contrast to anti-CD20, anti-CD19 depletes not only mature B cells but also short- and long-lived CD138^+^ plasma cells (130). However, CD1d^hi^ CD5^+^ regulatory B cells showed some resistance to anti-CD19-mediated depletion, which was not related to decreased CD19 expression (130). Together, these observations suggest that while anti-CD9 may reduce the B cell-related immune response, it may also spare some regulatory mechanisms (Figure 1). This may have a positive effect on autoimmune diseases but might favor the onset of opportunistic infections.

## Conclusion

The role of B cells in JCV infection and PML is likely more complex than initially thought. Indeed, on the one hand, B cells represent a potential reservoir for JCV and may disseminate the virus to the CNS while, on the other hand, they likely play a regulatory role in the immune response that controls JCV infection. The role of the humoral response in the control of JCV remains to be clarified but is probably less important than the T cell response. The association between rituximab and PML suggests that B cells may help to control JCV infection through functions other than antibody production. B cells secreting Th1-type cytokines such as IFN-γ probably enhance the Th1 response and thereby help to establish effective CD8 T cell activity against JCV. In addition, Treg responses are enhanced in B cell-depleted human and mouse models. These Treg responses could be induced by post-rituximab repopulating B cells, which could be predominantly IL-10-producing cells. A better understanding of the complex relations between JCV and B cells may have significant implications for the prevention and treatment of PML.

## Conflict of Interest Statement

The authors declare that this review was written in the absence of any commercial or financial relationships that could be construed as a potential conflict of interest.
